# Providing Sports Venues on Mainland China: Implications for Promoting Leisure-Time Physical Activity and National Fitness Policies

**DOI:** 10.3390/ijerph17145136

**Published:** 2020-07-16

**Authors:** Kai Wang, Xuhui Wang

**Affiliations:** 1 College of Landscape Architecture and Arts, Northwest A&F University, Yangling 712100, China; fywxuh@nwafu.edu.cn; 2 Centre for Urban Research, RMIT University, Melbourne, VIC 3001, Australia

**Keywords:** National Fitness Programs, per capita area, school sports facilities, urban parks, rural sports venues

## Abstract

Leisure-time physical activity (LTPA) has been well documented as having substantial health benefits. The 2014 Chinese Fitness Survey Report stated that a lack of physical activity (PA) spaces is the most important non-human factor, leading to 10% of leisure-time physical inactivity in people aged 20 and above. We investigated the provision of sports venues in China and discussed the development of sports venues and national fitness policies in the context of promoting LTPA and public health. We analyzed information from China’s most recent sport venue census, the Sixth National Sports Venues Census, conducted in 2013. The number of sports venues increased between 2000 and 2013, with an inflection point around the year 2008. At the end of 2013, there were 12.45 venues for every 10,000 residents, and the per capita area was 1.46 m^2^. However, numbers were still small compared with the United States and Japan. The percentages of full-time access, part-time access and membership venues were 51.5%, 14.3% and 34.2% respectively. Only half of sports venues were fully open to the public, meaning that the realized number and area per capita could be even lower. A lack of sports venues forces people who want to engage in PA to occupy other urban spaces that are not planned and designed for PA. Urban parks had 119,750 fitness station facilities (3.32% of the total), and 2366 urban fitness trails (19.24%), with a combined length of 6450 km (32.91%). On average, urban and rural areas had 13.17 and 10.80 venues per 10,000 persons, and 1.83 m^2^ and 0.97 m^2^ per capita. The urban-rural gap in sports venues exactly embodies some aspects of the “urban-rural dual structure” in China’s society. Measures to promote PA should focus on new and existing sports venues. In the policy making process, Chinese governments need to pay attention to the potential impact of related, external factors such as the gap between the urban and the rural and the potential advantage of indoor venues against summer heat and air pollution.

## 1. Introduction

Globally, more than 1.4 billion adults over the age of 18 did not reach the recommended levels of physical activity (PA) in 2016. According to the first study of worldwide trends in insufficient PA, this increases their risk of contracting the world’s major non-communicable diseases (NCDs), such as hypertension, cardiovascular disease, type 2 diabetes, dementia and some cancers [1]. As one of the most populous countries and a typical upper middle-income country, mainland China (hereafter “China”) faces a prevalence of NCDs and physical inactivity. Based on the Global Cancer Statistics 2018, China leads in cancer incidence and mortality rates in the world, and around one in five cancer patients come from China [2]. In 2017, over 114 million adults with diabetes lived in China (one in every 10 adults had diabetes), which was more than one-fourth of diabetes patients worldwide [3]. NCDs account for 88% of mortality in China, causing more than 70% of the total disease burden [4]. Zhang and Chaaban [5] suggest that physical inactivity contributes to an increased risk of five major NCDs (i.e., coronary heart disease, stroke, hypertension, cancer and type 2 diabetes), which in China constitute between 12% and 19%, and which alone account for more than 15% of the medical and non-medical yearly costs of the principal NCDs.

PA can be divided into leisure-time, occupational, transport-related and domestic activity categories. Occupational and domestic PA comprises the vast majority of PA among Chinese adults, while the amount of leisure-time and transport-related PA is very small in comparison [6]. A sharp decline in PA levels was observed by the China Health and Nutrition Survey using repeat measures and largely driven by a reduction in occupational and domestic PA. Transport-related PA, such as walking and cycling to and from work, has also sharply declined due to the skyrocketing car and e-bike ownership in China. The proportion of bicycle trips in Beijing dropped from 62.7% in 1986 to 11.3% in 2014 [7]. Bicycle lanes and sidewalks have been routinely compressed in order to tackle congestion problems caused by high car use. The increase in traffic injuries is a serious threat to the safety of pedestrians and cyclists. Wang et al. [8] investigated road traffic mortality in China from 2006 to 2016 and found pedestrians to be the most vulnerable road users. An in increase in sedentary jobs and an increased reliance on motorized transport have made leisure-time physical activity (LTPA) more important in fulfilling recommended PA levels [9].

LTPA has been well documented as having substantial health benefits, including improving physical wellness, lowering stress and depression, and leading to faster healing from medical conditions (also called recreational therapy) [10,11]. Although LTPA increased from 2.2 MET hours/week in 1991 to 4.8 MET hours/week in 2009 for Chinese adults, it was at 11.9 MET hours/week in 2009 for American adults and 14.8 MET hours/week in 2005 for British adults [12]. Additionally, this gap is projected to further widen in the future. The 2014 Chinese Fitness Survey Report (CFSR) states that the lack of PA spaces is the most important non-human factor, leading to 10% of leisure-time physical inactivity for people aged 20 and above [13]. The report also shows that about 34% of people aged 20 years and older who participate in LTPA use public sports venues, and another 12% use workplace or community sports venues that are only open to specific people. Sports venues play a vital role in promoting LTPA, especially public venues that are of no or low cost [14].

Chinese society has attached great importance to the development of sports venues. One key target of the Healthy China 2030 Plan and the National Fitness Programs is to enhance the growth of sports facilities. Since the founding of the People’s Republic of China in 1949, much effort has been made to build sports venues [15]. However, the insufficient resources of sports venues still restrict the proportion of adults who meet the minimum LTPA recommendation. In addition to the low per capita supply of sports venues, other characteristics of sports venues have a significant impact on the national levels of LTPA [16]. These characteristics include number and area, distribution in different networks, opening status, weekly visits, types of sports venues, sports venues in urban parks and urban-rural divide. Nevertheless, there have been few studies conducted on the provision of sports venues in China and on their impacts on LTPA and national fitness policies. Therefore, the purpose of this study was to investigate the provision of sports venues in China, analyze the pertinent characteristics of sports venues associated with LTPA and discuss the development of sports venues and national fitness policies in the context of promoting LTPA and public health. The findings of this study will be useful in clarifying the gap between the provision of sports venues and national LTPA targets, which could support decisions on upgrading existing sports venues and the size, type and function of new sports venues, while also preparing proposed investments.

## 2. Methods

### 2.1. Data and Subjects

The Sixth National Sports Venues Census (NSVC), China’s most recent one, was conducted in 2013. It included 84 types of sports venues (e.g., basketball courts, fitness stations and small-area sports courts) in various networks, industries and ownerships in China. The General Administration of Sports and the Ministry of Education led the national census. This census was divided into four hierarchical levels (national, provincial, prefecture and county), with data aggregated from the smallest to the largest spatial scale. Additional census details were obtained from the Data Compilation of the Sixth NSVC [17].

Sports venues refer to sports facilities dedicated to sports training, competitions and fitness activities, including functional affiliated buildings that are required for this purpose. Sites that are temporarily used for sports activities, such as conference rooms, auditoriums, warehouses, etc., are not included, and nor is construction. Major sports facilities used in this study included basketball courts (an outdoor stadium with fixed baskets for training and fitness, a minimum competition area 28 m long by 15 m wide and a surrounding, 2-m buffer extending from the competition area), fitness stations (facilities occupying small areas in communities, villages, parks, green areas, etc., consisting of a collection of outdoor fitness equipment, which are economical, practical and can be used free of charge), table tennis courts (an outdoor venue with fixed tables for playing table tennis, with a site area not under 40 sq. m), small-area sports courts (an outdoor stadium with a ring runway between 200 and 400 m), table tennis rooms (indoor sports venues for table tennis sports training and fitness use, at least 192 sq. m. Venues with temporary equipment and facilities in conference rooms, restaurants, auditoriums and corridors were excluded), track and field grounds (an outdoor stadium with a 400-m circular runway, no fixed stands or less than 500 seats), stadiums (a sports building with more than six standard 400-m runways, a soccer field in the center of the venue, a fixed grandstand and no less than 500 seats) and urban fitness trails (built in urban communities, residential areas, cultural and sports plazas, street gardens, waterfronts, parks and roadside green belts, free circular or non-circular trails open to the public for fitness).

The opening status of sports venues included membership (not open to the whole society, e.g., the sports venues of schools, enterprises and institutions are only open to the teachers and students of the school or the employees of the unit), part-time access (open to the public less than 8 h per day) and full-time access (open to the whole society every day for more than 8 h).

The criteria for regular PA in Chinese adults include three or more PA events per week of at least 30 min of moderate-intensity PA. The target heart rate during moderate intensity activities is about 64–76% of the maximum heart rate (The accepted method for obtaining the maximum heart rate is an estimation based on an individual’s age subtracted from 220) [18].

### 2.2. Analysis

Two categories of data were selected to describe the provision of sports venues. One category contained the property information of sports venues, i.e., number, area, type, opening status and weekly visits. The other was related to the ownership and location information of sports venues. We chose differences in sports venues between 2000 and 2013 to analyze past trends in the venue number and area. The number and area of sports venues were investigated in three networks (sports, education and others), including the corresponding opening status and weekly visits for publicly accessible venues. We sorted and analyzed the top five types of sports venues in number and area with full-time access status, venues in urban parks and the urban-rural divide in both outdoor and indoor venues. These data were compiled to calculate accessibility ratios and per capita use and to chart trends over time. The data analysis was conducted in Microsoft Office Excel 2016. All data were entered by two research assistants to ensure the initial data accuracy. Descriptive statistics were calculated.

## 3. Results

### 3.1. Trends in Number and Area of Sports Venues

**Figure 1:**
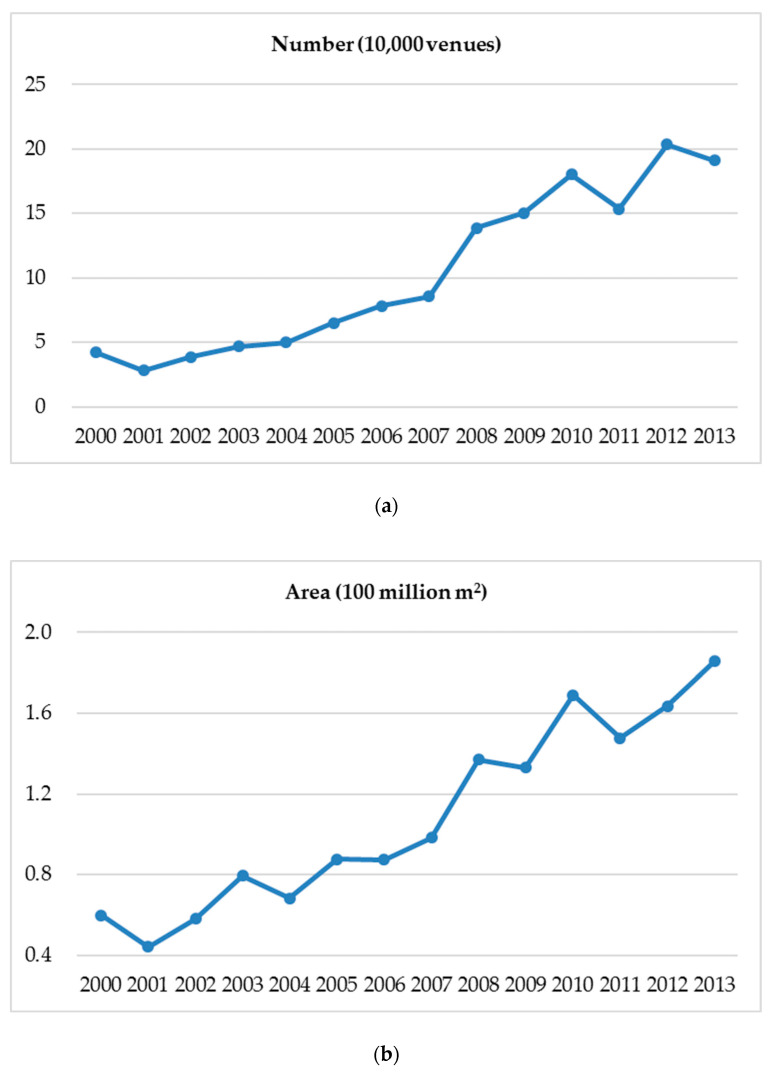
Annual growth in (**a**) number and (**b**) area of sports venues between 2000 and 2013.

The number of sports venues increased constantly between 2000 and 2013, with an inflection point around the year 2008 in which the annual growth in both number and area suddenly increased (Figure 1a,b). At the end of 2013, there were 1.69 million sports venues, and the venue area was 1.99 billion m^2^, including 0.17 million indoor venues and 1.52 million outdoor venues covering an area of 0.06 billion and 1.93 billion m^2^, respectively. There were 12.45 venues for every 10,000 residents, and the per capita area was 1.46 m^2^.

### 3.2. Sports Venues in Different Networks, Their Opening Status and Weekly Visits

Sports venues were distributed disproportionately across the sports network, education network and others (Table 1). More than 90% of venues were included in the education network and others. Under the education network, middle and elementary schools comprised 34.52% of the total venue number and 46.53% of the area.

In total, the percentages of full-time access, part-time access and membership venues were 51.5, 14.3 and 34.2%, respectively (Table 1). The sports network and others had 64.4 and 80.8% of full-time access venues. However, the education network had 68.3% of membership venues. Middle and elementary schools with a large number of sports venues had only 7.1 and 23.4% of full-time and part-time access venues. Among 1.08 million publicly accessible venues, 85.1, 11.7 and 3.2% of venues had less than 500, between 500 and 2500, and more than 2500 weekly visits, respectively.

### 3.3. Types of Sports Venues with Full-Time Access Status

The top five types of sports venues in number and area are shown in Table 2 and Table 3. Basketball courts were the most popular venues, both high in number and area (36.31% and 18.34%). Fitness stations had the second highest number (22.41%), and small-area sports courts covered the largest area (22.68%), and the percentages of full-time access were over 90%.

### 3.4. Sports Venues in Urban Parks

In 2013, there were 21,013 sports venues in 12,401 urban parks, with an area of 367,962 ha, i.e., 1.69 venues per park and 6.37 venues per 100 ha park area. Urban parks had 119,750 facilities with fitness stations (3.32% of the total), and 2366 urban fitness trails (19.24%) with a total length of 6450 km (32.91%).

### 3.5. Sports Venues in Urban and Rural Areas

In total, 58.62% and 68.60% of the number and area were located in urban areas, and 41.38% and 31.40% were in rural areas (Table 4). In terms of indoor venues, however, urban areas had 82.50% and 91.53% in number and area, and rural areas only had 17.50% and 8.47%. On average, urban and rural areas had 13.17 and 10.80 venues per 10,000 persons, and 1.83 m^2^ and 0.97 m^2^ per capita.

## 4. Discussion

This study represents the first analysis of the pertinent characteristics of sports venues associated with LTPA in China. The number and area of sports venues are the fundamental indicators for the overview of the characteristics. Both indicators increased dramatically between 2000 and 2013. The turning point occurred around the year 2008 when the Summer Olympic Games were held in Beijing, China. However, numbers were still small compared with the United States and Japan [19]. Every Japanese resident had 19 m^2^ of venue space on average in 2010 [20], but the Chinese per capita area was only 1.46 m^2^ in 2013. China’s Sports Development Five-year Plan is the national guideline for the growth goal of sports venues. The goals of the 12th Five-year Plan (2011–2015) were more than 1.20 million venues and 1.5 m^2^ per capita by the end of 2015. There were 1.69 million sports venues at the end of 2013, and the area per capita reached 1.5 m^2^ at the end of 2014 [21]. The new goal of the 13th Five-year Plan (2016–2020) was 1.8 m^2^ per capita. By 2030, the Healthy China Initiative aims to obtain 2.3 m^2^ per capita of sports venues. Indeed, a lack of sports venues can exacerbate use conflicts and potentially lead to social issues in China. For example, a group of elderly square dancers fought with young basketball players for the use of a basketball court in Luoyang city, in central China’s Henan province. Local police were finally called to stop the fight [22]. Media reported that similar incidents also happened in Fujian Province and Shanghai. A study led by the Central China Normal University found that Chinese teenagers’ increasing use of internet gaming can possibly be related to the low per capita area of sports venues in the country [23]. It is urgent to find paths for the sustainable development of sports venues in the context of China’s increasingly tense people-land relationship.

The opening status of sports venues can limit residents’ utilization of PA spaces. Our findings show that only half of sports venues were fully open to the public, meaning that the realized number and area per capita can be even lower. A lack of sports venues forces people who want to engage in LTPA to occupy other urban spaces that are not planned and designed for LTPA. For example, fast-walking groups have become a popular phenomenon that includes dozens, sometimes hundreds, of urban residents who use roadways to walk for exercise. Unfortunately, in 2017, a taxi crashed into a fast-walking group, causing one death and two injuries [24]. However, it is worth noting that 85% of publicly accessible sports venues received less than 80 daily visits, and only 18.4% of adults had access to the public sports venues, while nearly 40% chose to use vacant places that were not PA-oriented to engage in LTPA, such as in public open spaces or along streets [13].

Sharing campus sports venues can increase public PA opportunities, especially for nearby neighborhoods and communities [25,26]. More than one third of the number of and almost half of the area of sports venues were located at middle and elementary schools. Nearly 80% of children and youths aged between 6 and 19 engaged in regular PA, while only 14.7% of adults aged 20 and above were active [13]. This is likely because about 70% of sports venues in middle and elementary schools were not open to the public. Adults are generally no longer affiliated to middle or elementary schools, and thus they become a public that has a limited access to PA facilities within middle and elementary schools. In China, a higher level of education can lead to a higher percentage of participation in regular PA [13]. In addition to an awareness of exercise, this trend is likely related to school PA facilities (including high schools and universities) because a higher level of education means a longer access to campus PA facilities where it is much easier to develop an exercise habit.

The concept of shared use has been treated as an efficient and effective approach since social ecological models were adopted to develop PA opportunities [27]. The Global Action Plan on Physical Activity 2018–2030, released by the World Health Organization to reduce physical inactivity worldwide, proposed measures to encourage and strengthen the policy of shared use of school facilities with the strategic objective of creating active environments. The US “Healthy People 2020” strategy also established a similar objective to increase access to PA spaces and facilities in public and private schools. Japan’s Basic Act on Sport clearly states that Japanese national and public primary and secondary schools’ sports facilities need to be open to community residents [28]. As early as 2009, National Fitness Regulations released by the State Council of China proposed that public schools should actively create conditions to open exercise facilities to the public. However, many issues have accompanied these openings, such as how to maintain student safety, responsibility for accidental injuries of users, paying for increased management costs, etc. Lei divided the risk types of opening school facilities into charge management, personal safety, property security, environmental safety and liability [29]. In 2017, the Ministry of Education and General Administration of Sport in China jointly issued Implementation Opinions on Promoting the Opening of School Sports Facilities to the Society, stating that schools are required to open exercise facilities to students in their free time and on public holidays. By 2020, schools that meet opening conditions should also improve their level of openness and efficiency. The Implementation Opinions provided guidance on opening hours, potential users, the charging standard and the security mechanism. Although some cities were indeed promoting the opening of school exercise facilities, e.g., Shanghai and Hangzhou, the media often reported that residents complained that they had difficulty entering schools to use exercise facilities. As of 2018, there were 18 national policies on the opening of school exercise facilities [30], but the effect of the policies has yet to be tested.

The types of sports venues should match the needs of residents’ LTPA. Of all the sports venues, basketball courts accounted for the largest number and the second largest area. The 2014 CFSR shows that the out-of-school PA programs for children and adolescents aged 6 to 19 years included active party games (22.2%), long-distance running (18.0%) and basketball (11.2%), while for adults aged 20 and above basketball was rarely played [13]. The number of fitness stations was over 20% of the total, and over 90% were open full-time to the public. The users of fitness stations are largely middle-aged and elderly [31]. Because the first fitness stations to have been built are now approximately 25 years old (the first was built in 1996 at the Tianhe Sports Center of Guangzhou), many problems related to maintenance and the changing needs of a growing population need to be solved. These include the location, site layout, management and maintenance of fitness stations [32,33]. Small-area sports courts had the largest area, but only 7.79% of them had a full-time access, largely because most of these were found in middle and elementary schools. Li investigated 32 elementary schools within the downtown area of Dalian and found that 84.4% had small-area sports courts [34]. If the policy of opening school exercise facilities to society is to be well-implemented, the opening ratio of small-area sports courts must greatly increase in order to meet the growing needs for activities of adults aged 20 and older whose most popular activities are fitness walking (54.6%) and jogging (12.4%) [13]. Urban fitness trails can be another ideal venue for these activities. There are 12,299 urban fitness trails (nearly 19.6 million meters) nationwide, and more than 95% of the trails have a full-time access. However, Lin and Qiu studied the urban fitness trails in Fujian Province and found that about 40% of the trails received less than 500 visitors per week. This was, in part, because the trail venues were often located in less-populated areas or were built long enough ago to not meet modern exercise needs [35]. The Implementation Plan for the Million-kilometer Fitness Trail Project released in 2018 strives to construct about 300 km of fitness trails in each county-level administrative unit by 2020 [36]. The city of Suzhou in Jiangsu Province has been working on the country’s first urban fitness trail system plan [37].

Urban parks in developed countries that mix physical structures and green spaces are considered to be ideal environments explicitly built to promote LTPA and public health [38,39]. On average, US residents visit local parks and recreational facilities 29 times annually, and 52% of park users who were surveyed said that PA is a key factor in their decision to access parks and recreational facilities [40]. However, the importance of urban parks in promoting LTPA has not been fully valued or has even been ignored in the development of Chinese sports venues [41]. Only about 1.3% of sports venues are located in urban parks that are usually open to the public for free. In contrast, one observational park-based PA study shows that more than 50% of Chinese park users are able to engage in moderate-to-vigorous PA [42]. Additionally, 26.4% of people appeal for the building of sports venues close to urban parks [13], indicating that the public has a strong willingness to conduct physical activities in urban parks. In China, park-based running has become part of the urban lifestyle (increasing development in the process) [43], and fitness trails in urban parks play a vital role in this trend. For example, Beijing’s Olympic Forest Park has become China’s most popular running park, mainly because of its 13-km long, 3-m wide and 10-cm thick plastic fitness trails. The number and length of urban fitness trails in urban parks were nearly 20% and 30% of the total at the end of 2013.

China’s society has long been in a state of “urban-rural dual structure”, and the urban-rural gap of sports venues exactly embodies some aspects of this duality [19]. The number and area of urban sports venues were 1.42 and 2.18 times those of rural areas, and the number and area of urban indoor venues were as high as 4.71 and 10.8 times those of rural areas. In terms of the per capita number and area, the difference was at 1.22 and 1.89 times for total venues, and at 4.06 and 9.31 times for indoor venues. There is still a clear gap between urban and rural areas, particularly for indoor venues. The health risks associated with exposure to air pollution likely outweigh the benefits of outdoor physical activities in China [44,45]. Because most indoor pollutants can be efficiently reduced or removed by air purifiers [46], indoor venues become ideal places for healthy exercise without the negative effects of air pollution. The paucity of indoor venues in rural areas deepens this urban-rural divide. Residents’ rates of regular PA in urban and rural areas were 22.2% and 14.3%, respectively [47], a huge difference (7.9%) closely related to the aforementioned gap. Therefore, the National Fitness Program of China (2016–2020) stresses and promotes the extension of basic public exercise services to rural areas. China has made significant progress since the Fifth NSVC in 2003, when only 8.18% of sports venues were distributed in rural areas.

Our study was limited in several ways. First, the data used in this study were from 2013, the year of the latest decadal national census. However, sports venues have changed dramatically since 2013. For example, by the end of 2017, the overall number of sports venues exceeded 1.96 million, and the per capita area reached 1.66 m^2^, according to a newly published report that only described the overall trend [21]. Second, detailed data on the use of sports venues was unavailable, such as users’ gender, age and socioeconomic status. In addition, the incompatibility of statistical indicators in the Sixth NSVC and the 2014 CFSR made it challenging to conduct extended analyses. Third, non-standard sports venue data were not available. These venues are mostly found in rural areas, and the growth in the number of rural residents who regularly participated in PA was higher than that of urban residents [13]. Since non-standard sports venues usually use a small area and their investment cost is only one third that of standard venues, they may be more suitable for large cities with limited land resources or crowded historical quarters. Finally, urban parks play an important role in LTPA, yet information on park-based venues was lacking.

## 5. Conclusions

Public sports venues are physical spaces that accommodate fitness for all and are a basic resource for national public health. Accordingly, promoting the construction of public sports venues has always been one of the key contents of numerous sports developments, as well as of national fitness and public health policies. Promotion measures for sports venues should focus on both new and existing resources. On the one hand, China’s society aims to effectively expand new fitness resources to ensure that the per capita area continues to increase, particularly increasing popular fitness venues such as multipurpose small-area sports courts and urban fitness trails. More public open spaces, such as city parks, and urban vacant places, such as old factories, warehouses, old commercial facilities, etc., should be retrofitted as sports venues. However, the NIMBY (Not in My Backyard) issue occasionally occurs when selecting the location of sports venues [48]. The administration needs to further revitalize existing resources and pay close attention to the use, management and upgrading of sports venues and facilities. In particular, it is important to ensure that public and school sports venues that meet open conditions can be fully open to local residents. Finally, in the policy making process, Chinese governments need to pay attention to the potential impact of related, external factors such as the gap between the urban and the rural and the potential advantage of indoor venues against summer heat and air pollution.

## Figures and Tables

**Table 1:** Distribution and opening status of sports venues in different networks.

	Number Ratio (%)	Area Ratio (%)	Full-Time Access	Part-Time Access	Membership
Sports network	1.43	4.77	15,656	4868	3798
Education network	38.98	53.01	56,135	153,072	451,314
Others	59.59	42.22	774,050	76,333	107,184
Total	100	100	845,841	234,273	562,296

**Table 2:** Top five types of sports venues in number.

	Number (10,000 Venues)	Ratio	Full-Time Access
Basketball court	59.64	36.31%	47.60%
Fitness station	36.81	22.41%	91.37%
Table tennis court	14.57	8.87%	47.04%
Small-area sports court	8.91	5.42%	7.79%
Table tennis room	4.87	2.97%	34.78%
Total	124.80	75.98%	

**Table 3:** Top five types of sports venues in area.

	Area (100 Million m^2^)	Ratio	Full-Time Access
Small-area sports court	4.42	22.68%	7.79%
Basketball court	3.58	18.34%	47.60%
Ground track field	1.69	8.67%	12.42%
Stadium	1.05	5.39%	7.02%
Urban fitness trail	0.59	3.03%	96.68%
Total	11.33	58.13%	

**Table 4:** Number and area of sports venues in urban and rural areas.

	Urban Area		Rural Area	
	Number	Area	Number	Area
Indoor venues	12.87	0.54	2.73	0.05
Outdoor venues	83.40	12.83	65.24	6.07
Total	96.27	13.37	67.97	6.12
Indoor venues	12.87	0.54	2.73	0.05
Outdoor venues	83.40	12.83	65.24	6.07
Total	96.27	13.37	67.97	6.12

Notes: Number in 10,000 venues; Area in 100 million m^2^.
